# A Search-and-Rescue Robot System for Remotely Sensing the Underground Coal Mine Environment

**DOI:** 10.3390/s17102426

**Published:** 2017-10-23

**Authors:** Jingchao Zhao, Junyao Gao, Fangzhou Zhao, Yi Liu

**Affiliations:** 1 Intelligent Robotics Institute, School of Mechatronical Engineering, Beijing Institute of Technology, 5 Nandajie, Zhongguancun, Haidian, Beijing 100081, China; jch_zhao@bit.edu.cn (J.Z.); fzzhao@bit.edu.cn (F.Z.); YiLiu@bit.edu.cn (Y.L.); 2 Beijing Advanced Innovation Center for Intelligent Robots and Systems, Beijing Institute of Technology, Beijing 100081, China; 3 Key Laboratory of Biomimetic Robots and Systems, Ministry of Education, Beijing Institute of Technology, Beijing 100081, China; 4 Key Laboratory of Intelligent Control and Decision of Complex System, Beijing Institute of Technology, Beijing 100081, China

**Keywords:** search-and-rescue, robot, remote sensing, coal mine, explosion-proof, manipulator

## Abstract

This paper introduces a search-and-rescue robot system used for remote sensing of the underground coal mine environment, which is composed of an operating control unit and two mobile robots with explosion-proof and waterproof function. This robot system is designed to observe and collect information of the coal mine environment through remote control. Thus, this system can be regarded as a multifunction sensor, which realizes remote sensing. When the robot system detects danger, it will send out signals to warn rescuers to keep away. The robot consists of two gas sensors, two cameras, a two-way audio, a 1 km-long fiber-optic cable for communication and a mechanical explosion-proof manipulator. Especially, the manipulator is a novel explosion-proof manipulator for cleaning obstacles, which has 3-degree-of-freedom, but is driven by two motors. Furthermore, the two robots can communicate in series for 2 km with the operating control unit. The development of the robot system may provide a reference for developing future search-and-rescue systems.

## 1. Introduction

Coal mine accidents are difficult to avoid as long as humans still conduct mining activities. With the development of mining technologies, large-scale mine accidents have been reduced due to precautions in recent years, however, coal mine disasters still happen frequently in China. Just from the years 2006 to 2016 in China, there were about 20 coal mine accidents, and about 889 people died. Especially in the Tunlan mine accident, 78 people were killed, and 114 rescued people were injured on 22 February 2006.

Yan Gao et al. [1] have studied the occurrences of gas explosions and causes in China and the United States. Generally speaking, coal mining activities will encounter a complex and volatile geological environment, so accidents cannot be avoided. Thus, the rescue technology should be increased for reducing the number of casualties in accidents. The coal mine environment is too dangerous to send in rescue teams after accidents, because there will be poisonous gases, debris, flooding, unstable structures and explosive vapors. Therefore, the rescue teams is in the most dangerous environment. If there is a mobile sensor in front of the rescuers for perceiving dangers, they will be much safer.

In recent years, mobile robots equipped with multiple sensors have been proposed to inspect coal mine accident sites for acquiring the necessary environmental information and transmitting this to rescuers via a reliable communication system. The most important task is obtaining the accident site images and gas information for rescuers. If there are no dangers, the rescuers can go forward and reach the robots, then operate the robot to go on the rescue. Once the risky environmental information is obtained during the rescue, it will warn the rescuers to make some preparations. Therefore, this will make the rescuers much safer.

It is difficult to design a search-and-rescue robot system, and researchers should solve many serious problems, such as explosion-proof and waterproof design, passing barriers, detecting gas information, collecting images and sounds, data transmission, and so on, in the coal mine. In recent years, some search-and-rescue robots were created for coal mine rescue, and several robots have obtained safety certificate approval for mine products.

**Figure 1:**
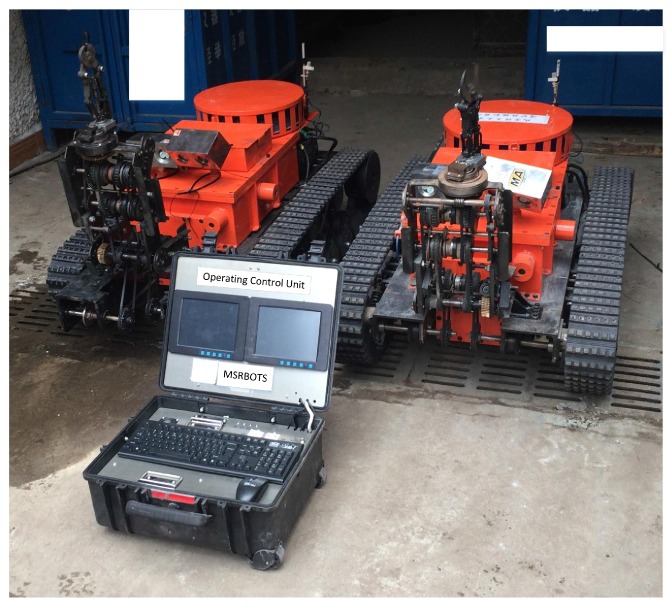
The search-and-rescue robot system used for remote sensing of the underground coal mine environment.

In this paper, a search-and-rescue robot system MSRBOTS used for remote sensing of the underground coal mine environment is introduced, which includes two robots and an operating control unit (OCU), and all parts in it are explosion-proof (shown in Figure 1). It has obtained approval for mining products certificated by Mining Products Safety Approval and Certification Center in China.

The MSRBOTS can keep working for 8 h. It collects 10 kinds of environmental information and transmits them to OCU by a fiber-optic cable (1 km long). The search-and-rescue robots in MSRBOTS can clean some barriers by their manipulators, such as cables and steel roll bars. In general, every joint of the manipulator should have a motor to drive, but every motor must have a steel explosion-proof shell in the coal mine, like the robot V-2’s manipulator [2]. This paper will introduce a novel manipulator; it is an 3-degree-of-freedom (including shoulder joint, wrist joint, scissor joint) manipulator with explosion-proof function and driven by two motors.

**Figure 2:**
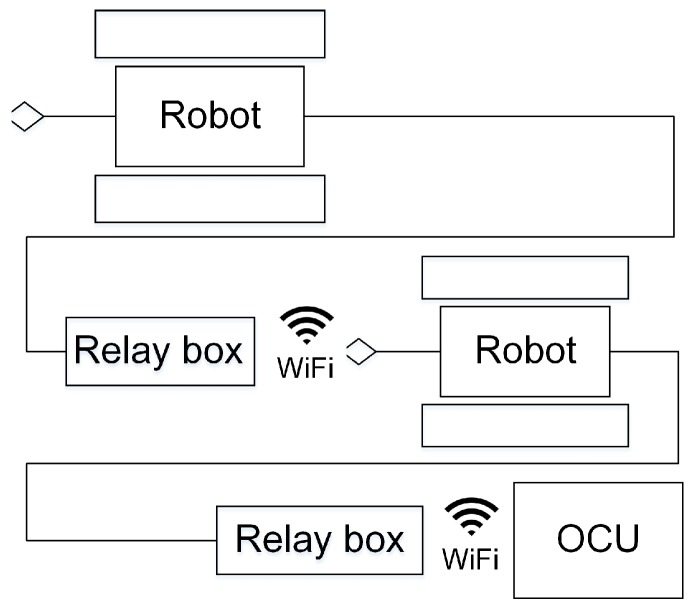
The illustration of robots communicating in series. OCU, operating control unit.

The robot by itself is not enough; it must also consider communication integration. The communication system of MSRBOTS is based on a network structure, and the devices of the MSRBOTS communicate with the OCU by a router housed in it. The communication between the OCU and the search-and-rescue robot is through a 1-km fiber-optic cable released by a passive autonomous spooling reel enveloped in a fiber-optic cable box. The operator can operate the robot to work in three modes: telecontrol mode, semiautomatic mode and automatic mode (rarely used). Especially, the two robots of MSRBOTS can communicate in series with OCU; thus, the longest communication distance can reach 2 km from the first robot to OCU shown in Figure 2.

This paper presents an overview of the MSRBOTS in recent years in Section 2. Section 3 presents the general structure of the MSRBOTS. The explosion-proof and waterproof design are shown in Section 4. Section 5 introduces the electric and control system of MSRBOTS. In Section 6, the testing, experiments and training carried out in Changzhou Testing Center of National Safety Approval and Certification Center and Tashan Coal mine of Datong Coal Mine Group are introduced, and according to the experiments, we trained the rescuers at the Rescue Training Scenes of the Datong Brigade of National Mine Emergency Rescue. Finally, the design and experiment are summarized. Section 7 is the conclusion.

## 2. Prior Works

Rescue missions in underground coal mines are different from rescuing on the ground; thus, researchers have done much work, and many successful applications of rescue robots have been developed and applied [3]. Particularly, the Center for Robot-Assisted Search-And-Rescue (CRASAR), directed by Murphy et al. [2], has completed many search-and-rescue tasks, including the 2001 World Trade Center rescue activity. Their research issues and design recommendations for rescue robots are as follows: (1) robots should have high mobility for traveling on complex terrain and slopes, and they can move on the ground with a rail line; (2) the communication system must be reliable, especially fiber-optic cables should be prevented from being tangled and breakage; (3) robots can clear obstacles in search-and-rescue missions; (4) the power capacity of batteries should be enough to allow the robots to work for a long time.

Kasprzyczak et al. [4] designed a pneumatic robot named GMRI . GMRI can work in methane and/or dust explosive gas environments, which is designed according to the M1 category in compliance with EN 50303. However, once the gas leaks, the roadway in the coal mine will be filled with new gas, and this may impact the surroundings. Several years later, they developed a new rescue robot platform named the Mobile Inspection Platform [5]; the robot is equipped with devices and sensors that allow the safe exploration and monitoring of coal mine environment. It is characterized by the following parameters: weight of about 1100 kg, maximal velocity 0.7 m/s, distance range 1000 m in the depth of excavation, dimensions of length 240 cm, width 115 cm, height 180 cm, supply 42 VDC. It is not possible to move the robot through an 80 cm-diameter hole in dams. The Ground Hog was developed by Carnegie Mellon University [6] for exploration and mapping of abandoned underground mines. It has explored kilometers of abandoned coal mines and performed many mapping tasks. It has produced high fidelity mine maps and models; however, the experiments could only be conducted in abandoned mines due to the lack of explosion-proof certification. Sandia National Laboratories [7] has created the Gemini-Scout to replace humans in the early stages of rescue and response during a mining accident. It is an intrinsically safe robot, and its mobility is very strong. Weidong Wang et al. [8] created a novel suspension system for a search-and-rescue robot named MINBOT-II, its characterized parameters are listed Table 1 compared with the MSRBOTS (mine search-and-rescue robot system); the detailed experiments and applications of MINBOT-II were discussed in their paper. Moczulski et al. proposed a new concept of a robot in 2014 [9]; their robot will mount a 3D imaging device and work in the coal mine, and this will be a big challenge. Furthermore, their robot can move through an 80 cm-diameter hole in fire dams.

These tasks summarize the robot performances in coal mine search-and-rescue missions and experiments. However, when it comes to the emphasis on researching the mobility of the robot, only the V-2 has been equipped with a manipulator. It can only catch an object by its clip, sometimes the robot needs to cut some cables and steel roll bars blocking it. The robots, in this paper, have a manipulator instead.

## 3. General Structure of MSRBOTS

The goal of MSRBOTS is obtaining environmental information; thus, the sensors in the search-and-rescue robot must perceive this information completely. It is not enough to rely on sensors. They should be sent into the accident sites. Therefore, the responsibility of search-and-rescue robots is bringing the sensors into dangerous areas under the operators’ control. Then, the search-and-rescue robot can be regarded as a mobile multisensor.

**Figure 3:**
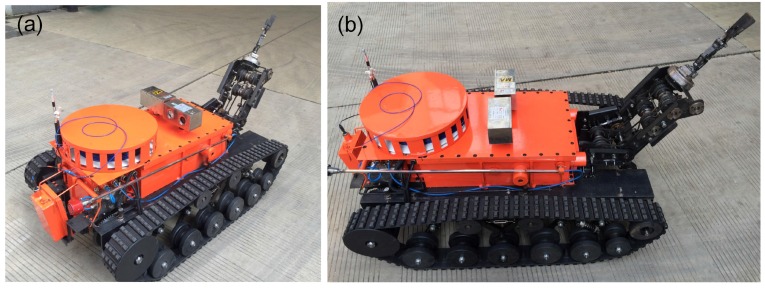
The coal mine search-and-rescue robot is shown in (**a**,**b**).

**Figure 4:**
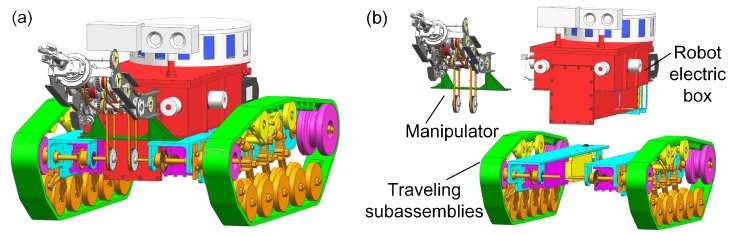
The coal mine search-and-rescue robot 3D model is shown in (**a**), the robot explosion structure is shown in (**b**).

The search-and-rescue robots are the key parts of the MSRBOTS (shown in Figure 3). The mobility of a tracked mobile robot is very important. Weidong Wang et al. [8] gave us some valuable experience in robot dynamic performance research. For search-and-rescue in the coal mine, a robot should realize the function of high mobility, a reliable communication system and explosion-proof and waterproof function. The MSRBOTS is explosion-proof and waterproof (shown in Figure 1), which includes two search-and-rescue robots and an operating control unit. Each robot is equipped with a robot electric box, a fiber-optic cable box, two traveling subassemblies and a manipulator (shown in Figure 3 and Figure 4). The parameters of the robots and OCU are listed in Table 1; furthermore, the parameters of MINBOT II are also listed for comparison.

The robot electric box is assembled with two traveling subassemblies, and the manipulator is fixed on the support beams of the two traveling subassemblies. Two gas sensors and a two-way audio are fixed on the robot electric box. The motors, for driving the robot and the manipulator, are assembled in the traveling subassembly beams, and both of them are enveloped in an explosion-proof and waterproof steel shell. As the robot is designed as modular, daily maintenance is facilitated. In this section, the general structure of MSRBOTS is introduced in detail.

### 3.1. The Robot Electric Box

**Figure 5:**
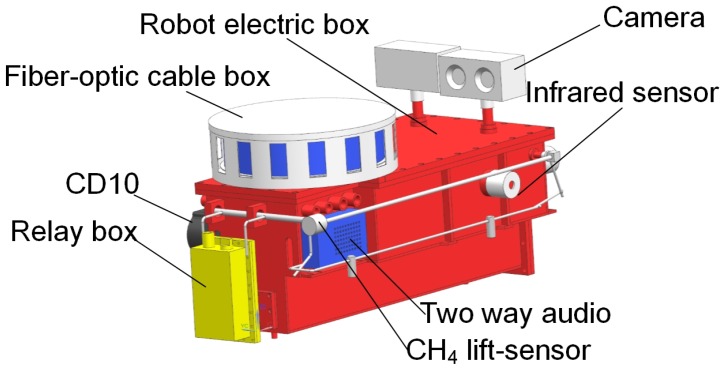
The structure of the robot electric box and fiber-optic cable box.

The robot electric box (shown in Figure 5) has four infrared sensors (ISs) for distance measurement, a CD10, a two-way audio and a CH_4_ lift sensor. Furthermore, the battery is in the bottom of the robot electric box. The four infrared sensors are integrated in the robot electric box; two of them are in the front, and the other two are in the left and right front position. The infrared sensors can assist the robot with avoiding impacting walls when traveling in the coal mine. The two cameras are integrated on the top of the robot electric box. The cameras are used for obtaining the video of the mine in real time. The two-way audio is placed on the right rear position of the end part of the robot electric box, which can be used for survivors to communicate with an operator. The CD10 is placed on the left rear position corresponding to the audio, which can detect 10 kinds of environmental information (including temperature, humidity, wind speed, air pressure and six kinds of gas concentration: CH_4_, O_2_, CO, H_2_S, CO_2_, SO_2_). Both the two-way audio and the CD10 are intrinsically safe devices.

In the coal mine tunnel, the methane (CH_4_) is not distributed on average along the vertical direction, and rescuers must clear the particular situation of the mine. Therefore, we create an intrinsically safe CH_4_ lift sensor, which is fixed at the end of a hollow tube driven by a motor for lifting up and down in the range of 0.4 to 1.7 m.

### 3.2. The Fiber-Optic Cable Box and OCU

Regarding the communication between OCU and robot though the fiber-optic cable, it is released via a passive autonomous spooling reel enveloped in a fiber-optic cable box.

**Figure 6:**
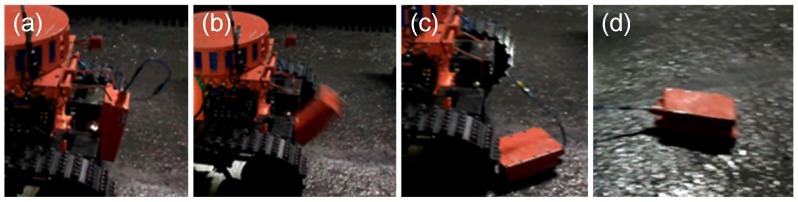
(**a**–**d**) show that the relay box was used up and dropped on the ground. (**a**) shows the relay box is at of the robot electric box. (**b**) shows the relay box is being released and falling down. (**c**) shows the relay box has dropped on the ground. (**d**) shows the relay box pulls the fiber-optic cable.

The fiber-optic cable box is fixed on the top of the robot electric box. The end of the fiber-optic cable is connected with the fiber-optic converter housed in the robot electric box, and the other end connects the other converter housed in the relay box. The relay box is a communication transmission station, and it can connect with a robot or the OCU by WiFi. The relay box stays in the back of the robot electric box, and it will be released and dropped on the ground automatically when operating the CH_4_ lift sensor (as can be seen in Figure 6). The link of the CH_4_ lift sensor drives a crank slider mechanism for releasing the relay box (as shown in Figure 5).

**Figure 7:**
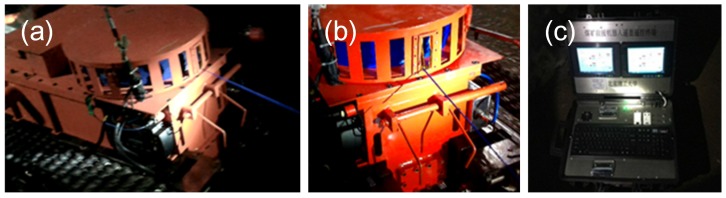
(**a**,**b**) show that the cable was pulled out from the fiber-optic cable box in different perspectives, and (**c**) shows that the OUC displayed the information received from the fiber-optic cable.

The fiber-optic cable reel spool on the plate can revolve around the fiber-optic cable box’s axis smoothly by a small pulling force (shown in Figure 7), so the relay box will pull the fiber-optic cable when the robot continues its task and goes forward. The length of the fiber-optic cable on a robot is 1 km, and the two robots can communication in series, so the longest communication distance is 2 km.

When the fiber-optic cable is used up, another robot begins to release the relay box and go on moving forward. Furthermore, the fiber-optic cable box is easy to disassemble, and the rescue team can carry several fiber-optic cable boxes to replace the one used up as the robot continues forward. The fiber-optic, having been released on the ground, could be used for the OCU communicating with the robot starting position.

### 3.3. The Traveling Subassemblies

The mobility of the robot relies on its traveling subassembly. Yu-tan Li et al. [10] have studied the motor dynamic performances in a test platform. Weijun Tao et al. [11] researched the motor during the robot traveling process. Both of them pointed to an important requirement: the traveling motor must have enough power and torque for climbing slopes in the coal mine. For the robots in this paper, two motors have been used for the manipulator, then two motors are adopted for back-driven method in the traveling subassembly.

**Figure 8:**
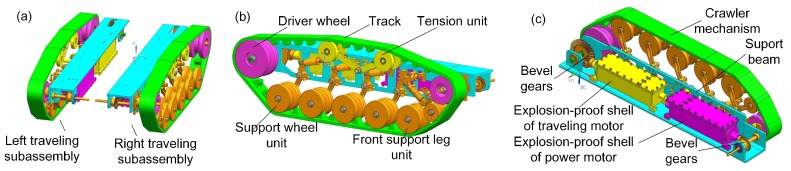
(**a**) shows the left and right traveling subassemblies, (**b**) shows the crawler mechanism and (**c**) shows the right traveling subassembly in detail.

The search-and-rescue robot has two traveling subassemblies, which are symmetrical assembled on the left and right side of the robot electric box by their support beams (shown in Figure 8). The left traveling subassembly consists of a support beam, a control motor, a traveling motor and a crawler mechanism. The beam as a connector organizes the other parts as a whole. The difference between the right traveling subassembly and the left is that the right one uses a power motor instead of the left’s control motor, and both of them serve the manipulator. In order to ensure safety in the coal mine, all motors are assembled in its explosion-proof and waterproof steel shell.

The power from the traveling motor transmits to the drive wheel through a pair of bevel gears. The robot uses crawler structures to travel on the ground of the mine. In order to make the robot stable when traveling, we adjust the position of the drive wheel on the back and higher than the support wheel units in the vertical. The robot is supported by 10 support wheel units with buffer springs assembled in two traveling subassemblies. When the robot is traveling on the ruins, they will effectively help it reduce the impact and vibration. In addition, a tension unit is designed for tensioning the track enough for crawler traveling stability.

**Figure 9:**
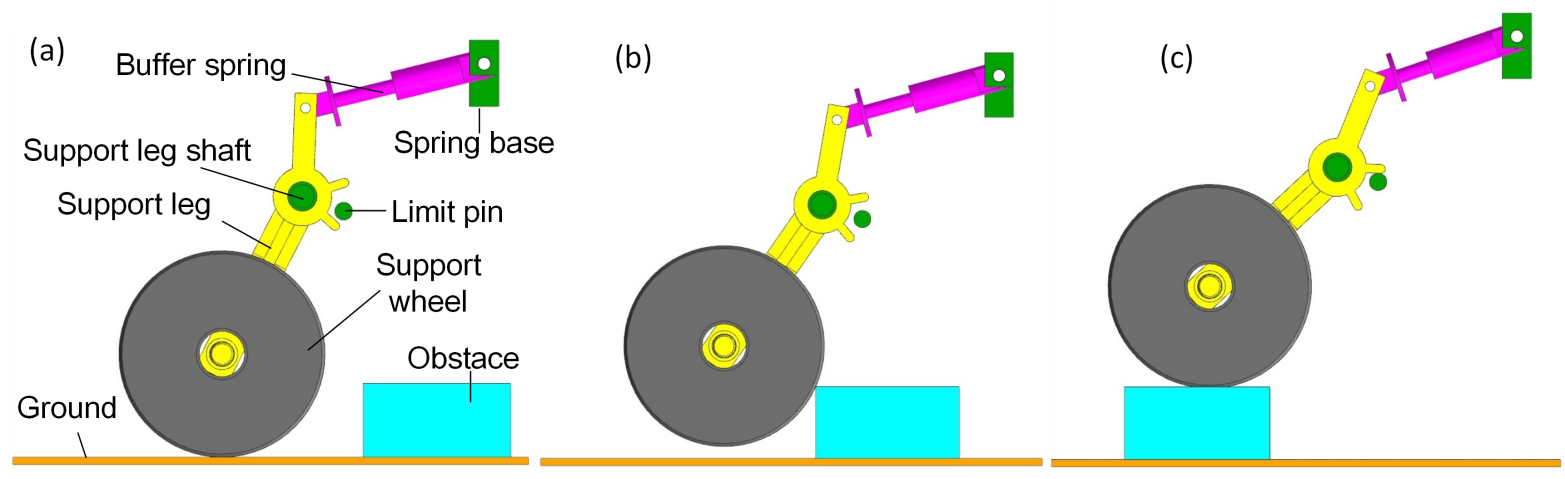
(**a**–**c**) show the support leg unit in the process of crossing the barrier. (**a**) shows the support wheel hasn’t hit the obstacle. (**b**) shows the support wheel is hitting the obstale. (**c**) shows the support wheel has been on the obstacle.

**Figure 10:**
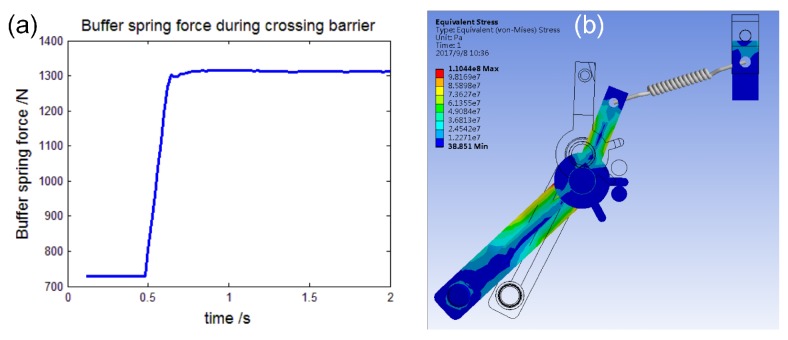
(**a**) shows the buffer spring force while passing the barrier. (**b**) shows the max equivalent stress contour of the support leg.

The support wheel unit is composed of a support leg, wheel and buffer spring. Figure 9a–c shows that it passes an obstacle. The leg is relative to the ground with a 60° angle to make the robot climb the obstacle smoothly. The buffer spring factor is 70 N/mm and will reduce the impact and vibration during the wheel climbing on the top of the obstacle. Figure 10a shows the force of the buffer spring during the obstacle passing process, and the max force is 1310 N. Each leg is strong enough to support a robot, and its FEM stress contour is shown in Figure 10b: the max equivalent stress is 98 MPa lower than the material’s yield stress of 235 MPa.

**Figure 11:**
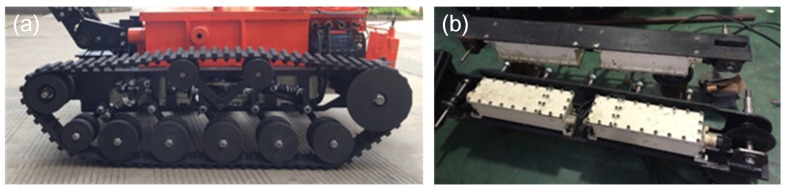
(**a**,**b**) show the left traveling subassemblies in detail. (**a**) shows the front view of the crawler mechanism. (**b**) shows the explosion-proof shells of motors.

The front support wheel unit has an auxiliary wheel, and it can help the crawler mechanism climb the barrier more easily, as shown in Figure 11a. Without it, the crawler mechanism will need more power to cross the barrier or it will never climb it.

### 3.4. The Mechanical Manipulator

**Figure 12:**
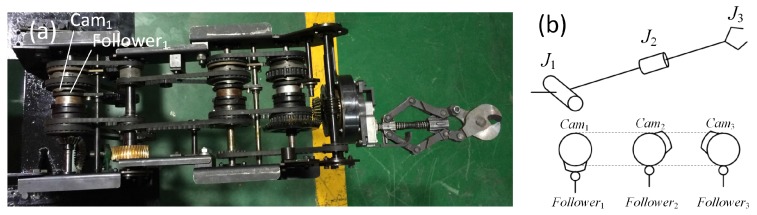
(**a**) shows the general structure of the manipulator, and (**b**) shows the illustration of the cam switch method for manipulator joints. $J_{1}$ represents the shoulder joint; $J_{2}$ represents the wrist joint; and $J_{3}$ represents the scissors.

A manipulator is necessary for a robot in some rescue and cleaning tasks, such as searching for survivors and clearing ruins in earthquakes or operating during dangerous tasks [12]. In this work, the most important requirement is that the manipulator be explosion proof, so a novel mechanical explosion-proof manipulator is designed (shown in Figure 12a). There are three joints in it, including the shoulder joint, wrist joint and scissors joint. The shoulder joint is used for lifting the manipulator; the wrist joint can rotate the scissors in the range of 0 to 360°; and the scissors can cut steel bars (diameter no more than 12 mm) and cables (diameter no more than 30 mm). Combined with the robot moving and swinging left and right, the scissors can catch any objects in the workspace.

Usually, every joint of the robot manipulator is driven by a motor, and this provides the robot the dexterity to perform the task. However, every motor should have an explosion-proof steel shell to work in the coal mine environment. If such a structure is adopted, the robot manipulator will be heavy and lack motivation. In order to solve this, we design a mechanical manipulator with two motors. One is regarded as the power motor and supplies power for the manipulator shoulder joint lifting, wrist joint rotating and scissors cutting. The other one, named the switch motor, switches the power from the power motor to the joint, which is specified to work by an operator.

The switch motor drives three pulleys synchronously, so their angular displacement remains the same at any time. Each pulley is the same and fixed with a cylindrical switch cam, the work area of switch cam is on its end face (shown in Figure 12b). Therefore, the work areas of three switch cams are distributed equally along the circumference. Thus, the interval is 120° between the work areas of the two adjacent pulleys. When the manipulator is driven lift, the power should be switched to the shoulder joint, then the operator drives the switch motor rotate and will not stop until Cam_1_ pushes its Follower_1_ down. When Follower_1_ is pushed down, it will push a slave clutch mesh to a master clutch, and the power from the power motor will be transmitted to the slave clutch. The slave clutch rotates synchronously with respect to the frame of the manipulator, so the manipulator is lifted by the power motor at this time. If any other joint is driven, the operator drives the switch motor to rotate the work area of the switch cam for pushing the corresponding follower down.

#### 3.4.1. The Shoulder Joint of the Manipulator

**Figure 13:**
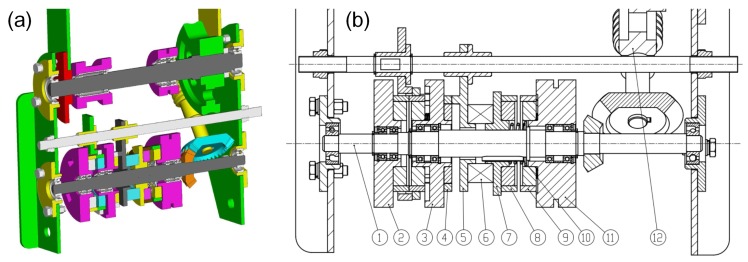
The illustration of the shoulder joint. (**a**) shows the 3D model section of the manipulator shoulder joint. (**b**) shows the manipulator shoulder joint in detail. The part number in this figure is used throughout the paper. ① Transmission shaft for transmitting the power to the shoulder joint. ②, ③ Switch pulleys, for switching power. ④ Switch cam, fixed with the switch pulley for pushing the follower side (Part ⑤). ⑤ Cam follower. ⑥ Thrust bearing, which is fixed with the follower and slider. ⑦ Slider, moving along the axial direction and rotating with the shaft (Part ①) synchronously. ⑧ Master clutch, fixed with the slider (Part ⑦). ⑨ Slave clutch, fixed with the drive pulley. ⑩ Guide key. ⑪ Drive pulley, driven by the drive motor. ⑫ Worm and worm gear.

**Figure 14:**
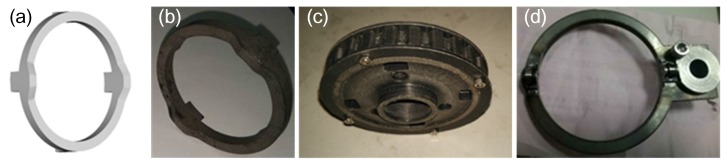
(**a**,**b**) show the 3D model and prototype switch cam (Part ④), (**c**) shows the switch pulley (Part ②) and (**d**) shows the cam follower (Part ⑤).

The power motor and switch motor are fixed in the support beams of the left and right traveling subassemblies, the rotation of the power and switch motors is transmitted through a pair of pulleys (as can be seen in Figure 4b). The slider component and worm component are used for the shoulder and wrist joint for switching the power to their joints. The scissors joint uses the slider component and pulley component to switch the power to cut/catch the object. All the parts of the slider component in the shoulder joint are shown in Figure 13. Which joint will obtain the power depends on whether the joint’s master clutch (Part ⑧) and slave clutch (Part ⑨) are meshed or not. The pulley, switch cam and its follower are shown in Figure 14.

**Figure 15:**
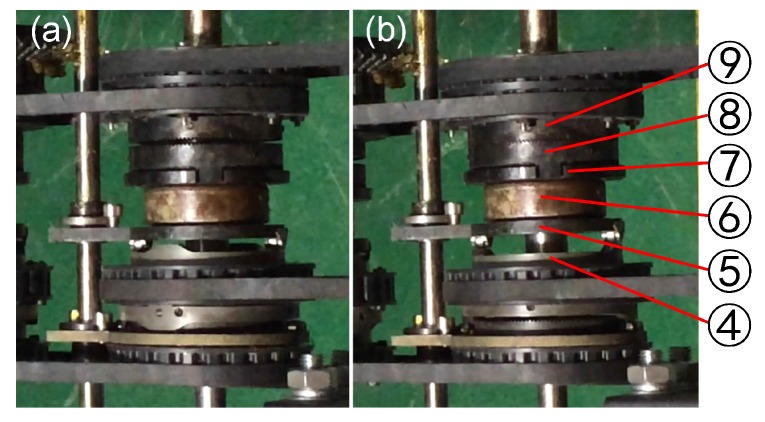
The slider assembly unit. (**a**) shows the master clutch (Part ⑧) and slave clutch (part ⑨) separate from each other. In (**b**), they have been meshed with each other.

The most important part of the slider component is the switch cam used for switching power to a joint. The follower (Part ⑤) will be pushed to the right by the work area of switch cam (Part ④). The follower (Part ⑤), thrust bearing (Part ⑥), slider (Part ⑦) and slave clutch (Part ⑧) are relatively synchronous in the axial and circumferential directions. When the follower (Part ⑦) is pushed by the work area of the switch cam (Part ④) to the right, the slave clutch (Part ⑧) meshes to the master clutch (Part ⑨), and this process is shown in Figure 15. Then, the power transmits to the transmission shaft (Part ①), which drives the worm (Part ⑧) to rotate by a pair of bevel gears. The worm gear is fixed with the next link of the wrist joint, so the manipulator is driven to lift by the power motor. The spiral angle is 4°; thus, the robot manipulator can hold its posture by the self-lock function of the worm component.

**Figure 16:**
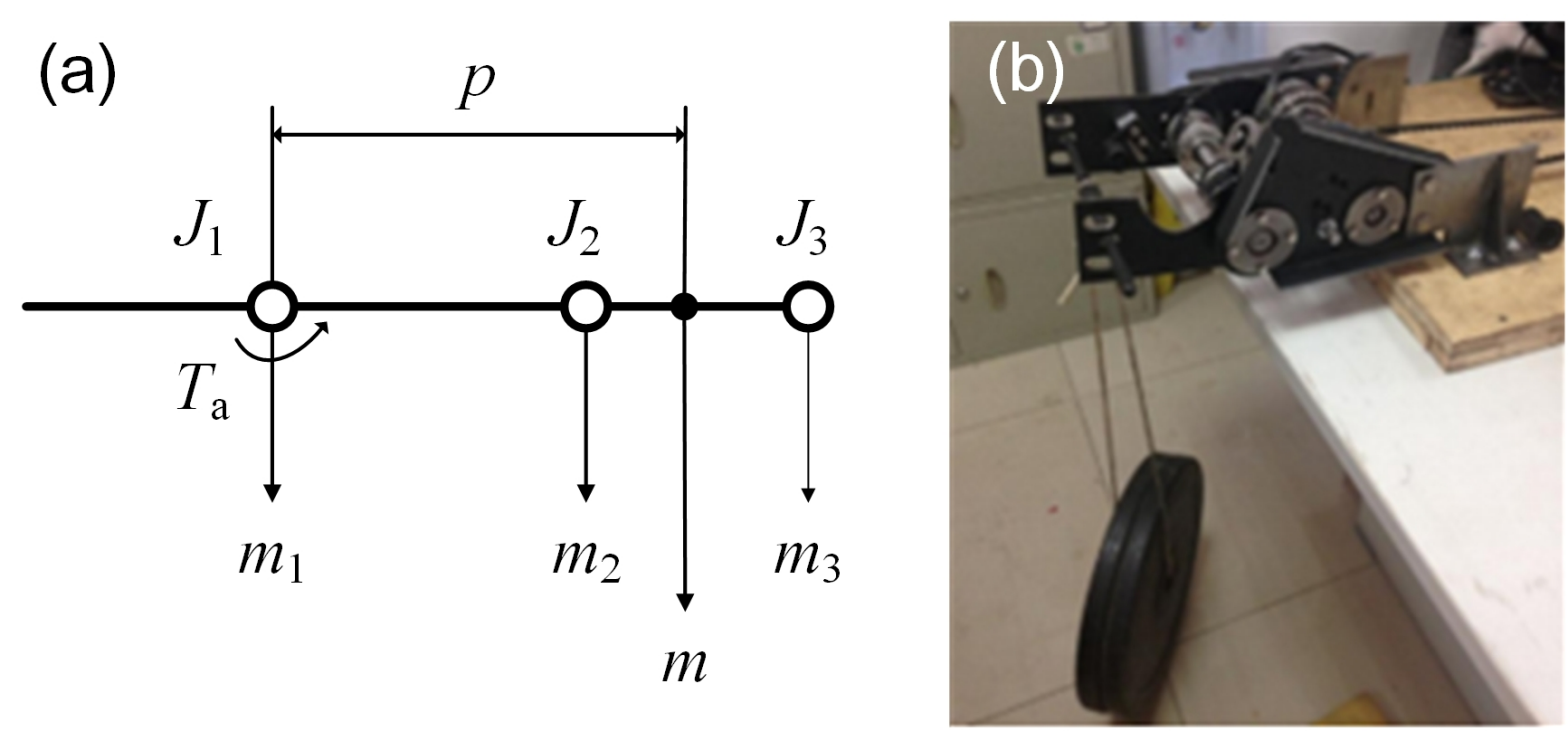
(**a**) shows the mass distribution of the manipulator; (**b**) shows the experiment of the shoulder joint.

The shoulder joint bears the largest load among the three joints, so its strength requirement is very important. The manipulator is 15 kg; the mass distribution is shown in Figure 16a. The shoulder can bear about 112.5 Nm (shown in Figure 16b). The detailed parameters of the manipulator are listed in Table 2. When $\theta_{a}$ is −20°, the scissors of the manipulator will touch the ground.

#### 3.4.2. The Wrist Joint of the Manipulator

**Figure 17:**
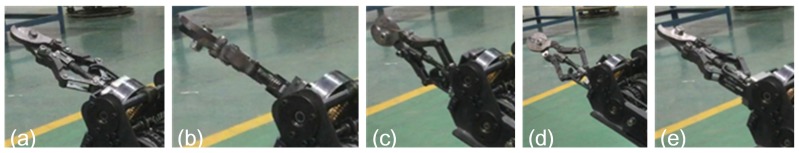
The rotating performance of the wrist joint assembly unit. (**a**–**e**) show the process of the scissors rotating 360° counterclockwise, which is driven by the wrist joint.

The wrist joint is used for adjusting the posture angle of scissors to point to the target for cutting or catching. The power switching for the scissors is also the same as the shoulder joint, but the power is transmitted by a pair pulleys from the transport shaft to the worm. In order to point to the target, the posture angle of the scissors should adjust to any angles on the circumference (shown in Figure 17), and they can also keep the posture angle of the scissors after the adjustment. Therefore, worm component is used because of its self-lock function.

#### 3.4.3. The Scissors of the Manipulator

**Figure 18:**
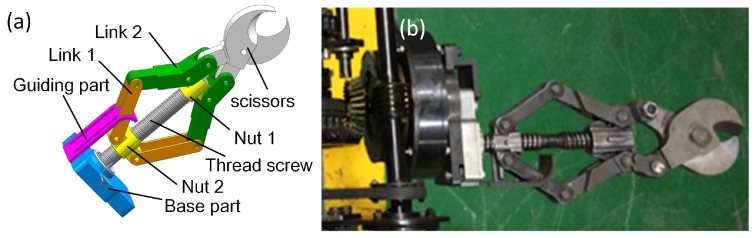
(**a**) shows the structure of the scissors of the manipulator; (**b**) shows the scissors’ prototype.

**Figure 19:**
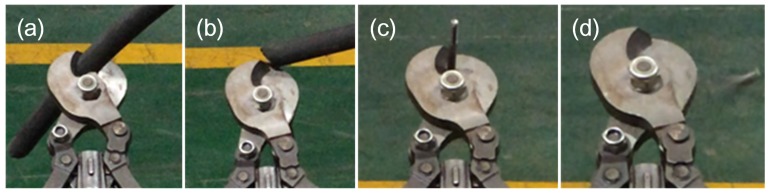
The cutting performance of the wrist joint assembly unit; (**a**,**b**) show scissors cutting cable; (**c**,**d**) shows cutting steel roll bar.

Similarly, the power switching for the scissors is also the same as the shoulder joint. Then, the reducer drives a thread screw to perform the cutting/catching work of the scissors. The structure of the scissors mechanism is shown in Figure 18a. The thread screw has two helix sections: the former is right-handed, and the other is left-handed. When the thread screw is turned to a clockwise direction, the two nuts will be close to each other, and the blades of the scissors will cut or catch a target. The power is transmitted to the reducer of the scissors from transport shaft by a pair of bevel gears (as can be seen in the left side of Figure 18b). The process of cutting out cables and steel roll bars is shown in Figure 19.

## 4. The Explosion-Proof and Waterproof Design for MSRBOTS

The explosion-proof technologies are mature both mechanically and electrically, and they have formed industrial standards. Many explosion-proof robots have been designed, and there are two kinds of techniques that are generally explosion-proof and intrinsically safe to enable a robot to fulfil explosion-proof regulations. Intrinsically safe devices can be exposed to the coal mine environment. The explosion-proof technique is a protection technique for electrical apparatuses that are not intrinsically safe. The MSRBOTS has obtained approval for mining products by Mining Products Safety Approval and Certification Center in China. Furthermore, the waterproof function is designed to be Ingress Protection Rating (IP) 67. grade by static sealing and dynamic sealing.

### 4.1. The Explosion-Proof Design for the Robot System

In MSRBOTS, the cameras, audio, CD10 and CH_4_ lift sensors are intrinsically safe. The other electric apparatuses must be housed in an explosion-proof box to ensure that these apparatuses do not initiate an explosion. All the design features in MSRBOTS about the explosion-proof design are according to the national standard GB3836, including the materials used being explosion-proof and flame retardant.

#### 4.1.1. The Explosion-Proof Design of the Mechanical Structure

**Figure 20:**
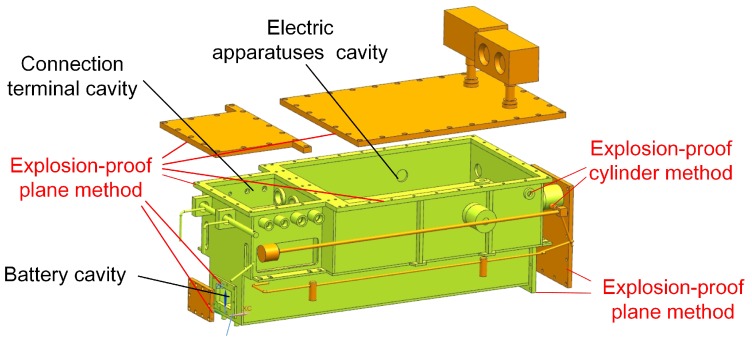
The mechanical explosion-proof method of the robot electric box.

**Figure 21:**
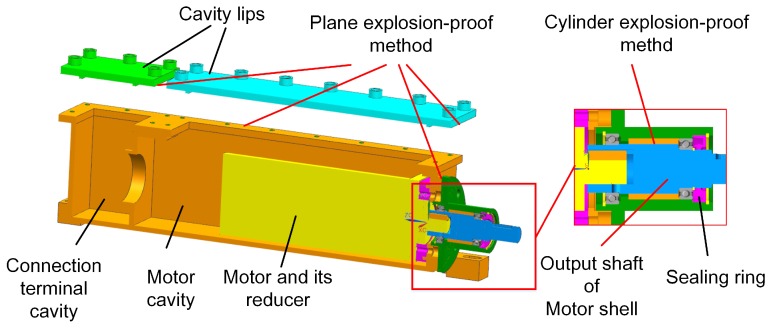
The mechanical explosion-proof method of the motor box.

The explosion-proof box in robots includes a robot electric box, a switching motor box, a power motor box and two traveling motor boxes. The plane explosion-proof method, cylinder explosion-proof method and gum-filling explosion-proof method are widely used in explosion-proof equipment, and they have been marked in Figure 20 and Figure 21 for the robot electric box and motor boxes, except the gum-filling explosion-proof method. The gum-filling explosion-proof method is used in the battery cavity, because the gum will occupy the capacity in the battery cavity for reducing the volume of flammable gases.

#### 4.1.2. The Explosion-Proof Design of the Electric System

The explosion-proof design of the electric system focuses on preventing the circuits from igniting the gas in the coal mine. Between the sensors and the control system, an explosion-proof isolation unit is designed for the electric system. Furthermore, some researchers tested the electromagnetic compatibility of the mine mobile inspection robot [13]. In MSRBOTS, all the electric components are housed in the robot electric box, relay box and OCU. There are three cavities in the robot electric box (shown in Figure 20), including the battery cavity, electric apparatuses cavity and connection terminal cavity. The connecting method between the cavities is according to the related explosion-proof national standard. The battery cavity is used to place the battery. There are some connection terminals in the connection terminal cavity used for arranging and outputting cables to the outside robot electric box. The communication and power supply between motors’ explosion-proof boxes and the robot electric box adopt cables, and the connection method also meets the related national standard. The relay box is also an explosion-proof steel shell; however, the communication between the robot electric box uses the fiber-optic cable, and it is not necessary to design an explosion-proof isolation unit. The electric apparatuses in OCU are all intrinsically safe, so the OCU does not need to be designed with explosion-proof features.

### 4.2. The Waterproof Design of the Robot

The protection grade of the robot water sealing requirements in coal mines has to be IP67. The sealing type in our robot can be divided into kinds: static sealing and dynamic sealing. For the static sealing method, the static waterproof (O ring) is used at the transition parts and connection parts. The static sealing and dynamic sealing surfaces in the robot electric box are all static sealing, except for the shaft for the CH_4_ lift sensor, but the O ring is still used for waterproof performance because the speed of its output shaft is very low. The output shafts use dynamic sealing, and other contacting surfaces use static sealing in motor boxes. For movable components, such as the power output shaft, a dynamic sealing method is utilized, as shown in Figure 21.

## 5. The Control System of MSRBOTS

### 5.1. The Electric System of MSRBOTS

The electric system of the coal mine rescue robot system includes the sensor system, battery, motion control unit, OCU and communication system.

The sensor system is used for detecting the environment in the coal mine and the status of the robot, including the CH_4_ lift sensor, CD10, two cameras, a two-way audio and battery management system (BMS). The CH_4_ lift sensor is an intrinsically safe sensor, works with full duplex, has a working voltage of 3 V and a work range of 0 to 100 vol%. The CD10 is characterized by the following parameters: the range of wind speed is 0.2 to 20 m/s, humidity is 0 to 100% RH, atmospheric pressure is 100 to 1400.0 hPa, temperature is −15.0 to 50.0 ${}^{\circ }$C.

The battery is managed by BMS and used for supplying steady electric energy for the electric system. The electric energy of the target robot can support the robot movement for 5 km.

**Figure 22:**
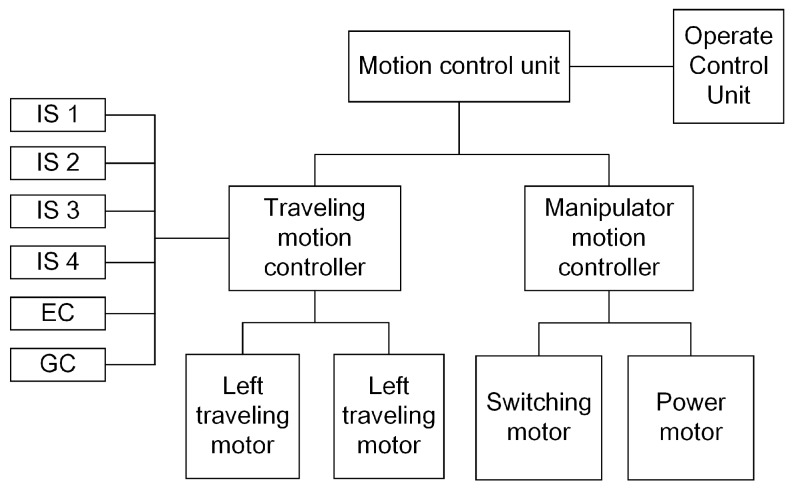
The frame of motion control unit.

The motion control system is a control unit (shown in Figure 22), and it can not only control the switching and power motors of the manipulator, but also the two traveling motors during movement. Furthermore, the motion control unit includes four infrared sensors (ISs), an electronic compass (EC) and a gyro (GY). The four infrared sensors are used for protecting the robot from hitting the wall. When any infrared sensor measures the distance from the wall or other obstacles as close, 0.02 m, the motion control unit will stop the robot and send warning messages to the OCU. The electronic compass and gyro are used to locate the robot position.

**Figure 23:**
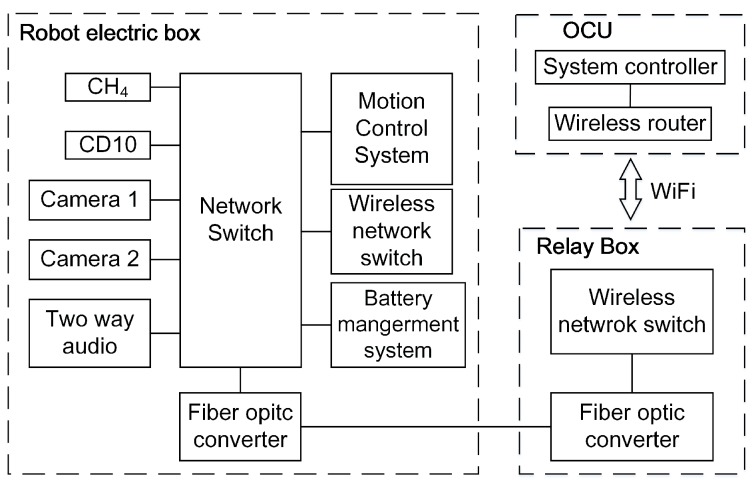
The frame of the robot communication system.

Communication in coal mines has been studied for many years [14,15,16,17]. Hussain et al. [18] researched the effect of the coal mine environment for the communication system. The function of the communication system is to achieve communication smoothly between the OCU and the robot, including robot motion commands, video, sound and the information obtained by the sensor system. It should transmit the messages to the robot sent by OCU and return the information that the OCU required. The communication system uses the fiber-optic cable to transmit the video, audio and messages unimpededly. The frame of the communication system is based on the network (shown in Figure 23), so every unit can communicate with other units in the system. Additionally, if any unit stops working, the other components of the control system will still work normally. The communication system includes a network switch (NS), two fiber-optic converters, a 1 km-long fiber-optic cable, two wireless network switches and a wireless router. The network switch organizes the apparatuses in the robot electric box as a whole. After many experiments, the network frame of the communication can ensure the unimpeded communication effectively. Furthermore, the two robots can communicate with each other by their wireless network switches for communication in series (shown in Figure 2). Furthermore, when the search and rescue robot finishes its mission, it can be controlled by the OCU without the optic-fiber cable. The OCU can communicate with the robot by the WiFi communication at this time. As Figure 23 shows, there is a wireless router in OCU, and a wireless switch is housed in the robot electric box. They will be connected with each other when they are close enough. Then, the robot can be controlled by the OCU directly without the help of the optic-fiber cable.

OCU is an interactive interface platform for operators to control whole MSRBOTS. The router, in the OCU, is the center of the communication system. OCU shows the robot status and coal mine environmental information for the operator and other rescuers and sends commands to the robot from the operator.

There is an explosion-proof computer in the OCU, and the application software used for controlling BSRBOTS is installed in it. The communication between the OCU and electric apparatus in the robot electric box (shown in Figure 23) is based on the TCP/IP protocol. A micro-controller (STM32) without the operating system is responsible for the motion control of the robot.

### 5.2. Human-Interaction

The OCU, as the platform for the human-interaction system, provides an interactive interface for the operator to control the system, including the software for the control system, two display windows, a microphone, a speaker, a mouse and a keyboard and other electric apparatuses, and all the apparatuses in OCU are intrinsically safe. Furthermore, the human-robot interaction system provides three control modes for the robot and an acceptable method for the robot localization in the coal mine. In order to make the robot complete search-and-rescue tasks successfully, good coordination of the robot movement and clearing tasks of the mechanical manipulator is very important. Therefore, the human-robot interaction has the responsibility of controlling the manipulator well.

In this section, we discuss an acceptable method for the robot localization in the coal mine at first. Then, we discuss the mechanical manipulation motion method for cutting and catching tasks. Finally, the robot motion method in the coal mine is discussed, and after some experiments in the coal mine, two kinds of practical motion control modes are adopted: telecontrol mode and semiautomatic control mode.

#### 5.2.1. Robot Localization in the Coal Mine

The position of the robot in the coal mine is important for rescuers to observe the environment [19,20]. Due to GPS being unavailable when the robot works in the underground coal mine, the electronic compass, gyro and two code plates of traveling motors are used to deduce the robot movement trajectory. During the movement, when the robot turns in place, it cannot avoid measurement errors from the electronic compass and gyro, because of the slipping between the robot track and ground. Then, the movement trajectory can be integrated with the characteristics of the environment in the video [21] obtained by two cameras and the drawings of the coal mine structure to determine the robot position. Although this method has many defects, it can help the rescue team localize the robot.

#### 5.2.2. The Manipulator Motion Method

**Figure 24:**
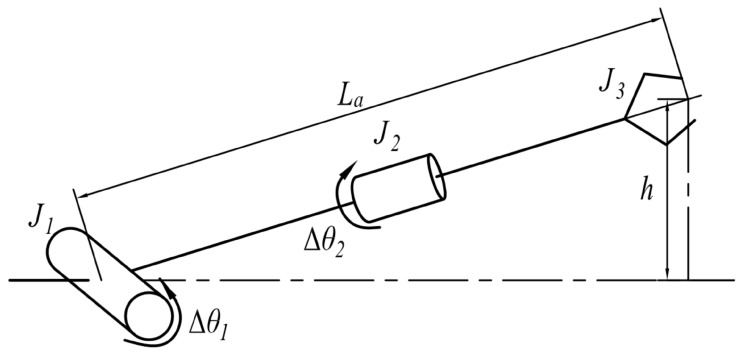
The illustration of the manipulator motion parameters. In the figure, $J_{1}$ represents the shoulder joint; $J_{2}$ represents the wrist joint; and $J_{3}$ represents scissors. $\Delta \theta_{1}$ and $\Delta \theta_{2}$ represents the rotation angle of the shoulder and wrist joint; *h* represents the current height of the scissors; $L_{a}$ represents the length of the manipulator.

The manipulator has three joints, which are driven by two motors, and only one joint is working at any time. In practice, for adjusting the manipulator posture to point to obstacles, the height and angle of the scissors should be adjusted correctly. The shoulder joint decides the height of scissors, and the posture angle of the scissors is controlled by the wrist joint. At the beginning, the operator should drive the shoulder joint to adjust the height of the scissors before adjusting the posture angle of the scissors. When the shoulder joint lifts an increment in height (Δh) relative to current height (*h*) of the scissors (shown in Figure 24), the controller will calculate the angle ($\Delta \theta_{pm}$) by which the power motor needs to rotate. The calculation of the rotation angle of the power motor is calculated in Equations (1) and (2):

(1) $$\Delta \theta_{1}=\arctan\left(h+\Delta h\right)-\arctan h$$

(2) $$\Delta \theta_{pm}=i_{J_{1}}\cdot \arcsin\left(\frac{h+\Delta h}{L_{a}}\right)-\arcsin\left(\frac{h}{L_{a}}\right)$$

where $i_{J_{1}}$ is the shoulder reduction ratio, which represents the transmission ratio from the power motor to the shoulder joint ($J_{1}$), and its value is 720.

When the operator drives the wrist joint, the rotation angle of the power motor is calculated in Equation (3):

(3) $$\Delta \theta_{pm}=\Delta \theta_{2}\cdot i_{J_{2}}$$

where $i_{J_{2}}$ is the wrist reduction ratio, which represents the transmission ratio from the power motor to the wrist joint ($J_{2}$), and its value is 480.

The manipulator motion controller controls the scissors joint to cut/catch and open. When the scissors cuts out targets, the motion control unit will stop to open automatically. If the scissors caught the target, the motion control unit will stop to hold the catching status. Both working processes are controlled by power motor controller based on current feedback. After many experiments, the suitable speeds for driving motors under different working conditions are obtained: (1) when the shoulder joint is lifted up, the power motor should be operated in a range of speed of 850 to 1100 rpm, and it should be below 600 rpm when it descends; (2) when the scissors joint is working, the power motor should be at the fastest speed.

#### 5.2.3. Robot Motion Method

Due to the robot being under remote control when it moves in the coal mine, it needs to brake in a timely manner to avoid hitting the wall and obstacles. Therefore, four infrared sensors in the robot electric box continue working to measure the distance from the robot to the wall or obstacle and send the feedback to the robot motion control unit for protecting itself automatically without the control of the OCU.

**Figure 25:**
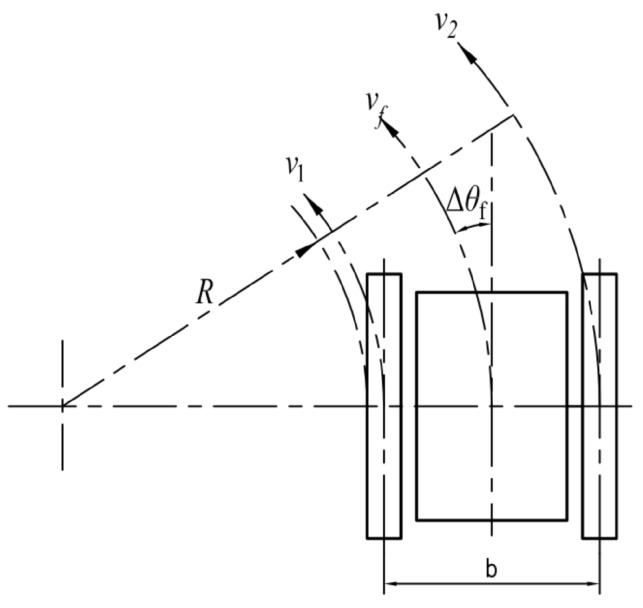
The illustration of the robot motion parameters. In the figure, *R* represents the radius of the robot when turning, $\Delta \theta_{f}$ represents the deflection angle relative to the current traveling direction, $v_{1}$ and $v_{2}$ represent the speed of the left and right traveling subassemblies, $v_{f}$ represents the speed of the robot and *b* represents the distance between the left and right subassemblies.

Because of the rugged roadway in the coal mine, remote control is very difficult [22], so operators need suitable control methods. There are three control modes for the robot system usually: (1) the telecontrol mode; (2) the semiautomatic control mode; (3) the automatic control mode. After some experiments, the telecontrol and semiautomatic control mode are preferred. When the robot is blocked by obstacles, the telecontrol mode can be adopted for clearing them. The semiautomatic control mode is used in simple situations, such as going straight or turning corner. The automatic mode is used in very ideal conditions, but it rarely appears in the coal mine. The illustration of the robot motion parameters is shown in Figure 25.

##### The Telecontrol Mode

The telecontrol mode is used in complicated environments to adjust the robot’s position slightly. In this mode, the deflection angle, the tracks’ displacement and the moving time are regarded as the control parameters for the robot to turn. The final control objects are the speeds of the left and right tracks. The tracks’ speeds are calculated as Equations (4) and (5).

(4) $$v_{f}=\frac{\Delta s}{\Delta t}$$

(5) $$v_{1}=-v_{2}=\frac{b\cdot \tan\Delta \theta_{f}}{\Delta t}$$

In practice, the robot turning in place is frequently used, but the speeds of the left and right tracks must coordinate well with each other for preventing the tracks from separating from the driver wheel. Finally, the relationship between the deflection angle and adjusting time to coordinate the speeds of the tracks have been summarized, and all the data of the relationship have been implanted in the control program, so the operator can extract them directly. In a complicated environment, the robot should adjust its body position at first and then adjust the posture of the manipulator.

##### The Semiautomatic Control Mode

Under the semiautomatic control mode, two control method are adopted effectively:

(1) The speed of the robot and the deflection angle ($v_{f}$–$\Delta \theta_{f}$ method) are regarded as the control parameters, and the speeds of the tracks are calculated in Equation (6):

(6) $$\left\{\begin{array}{c} v_{1}=v-\frac{b\cdot \Delta \theta_{f}}{2\Delta t}\\ v_{2}=v+\frac{b\cdot \Delta \theta_{f}}{2\Delta t} \end{array}\right.$$

(2) We use the robot speed and the turning radius ($v_{f}$–$\Delta \theta_{f}$–*R* method) to control the robot to turn at the corner in the coal mine. The speeds of tracks are calculated in Equation (7):

(7) $$\left\{\begin{array}{c} v_{1}=v_{f}\frac{2R-2b}{2R-b}\\ v_{2}=v_{f}\frac{2R}{2R-b} \end{array}\right.$$

Under the semiautomatic mode, no matter whether going straight or turning around in the coal mine, the robot can go forward as quickly as is possible, so it will save some time in the rescue process.

## 6. Test and Experiment

In the first part, the test of MSRBOTS is introduced. It was tested at the Mining Products Safety Approval and Certification Center Changzhou site. In the second part, the MSRBOTS was tested in the Tashan Coal mine of Datong Coal Mine Group for research. In the last part, the rescuers were trained to use MSRBOTS in the Rescue Training Scenes of the Datong Brigade of National Mine Emergency Rescue.

### 6.1. Testing at the Center of National Safety Approval and Certification Center

The MSRBOTS was tested at the Safety Approval and Certification Center Changzhou site, and the test content included mobility, communication, battery supply capacity, environmental information collecting, explosion-proof and waterproof ability. Parts of the test results are listed in Table 3, and the test results of MINBOT II are also listed for comparison.

**Figure 26:**
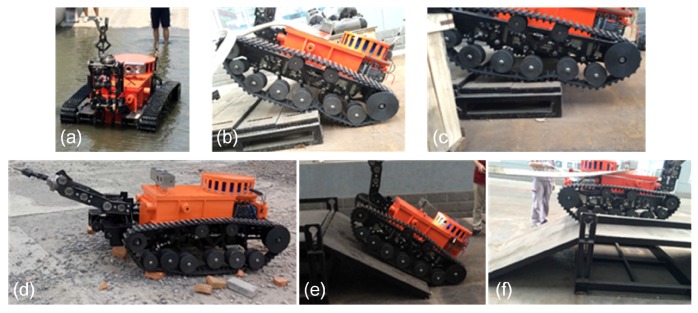
Test at the Changzhou site of Center of National Safety Approval and Certification. (**a**) shows that the robot was traveling and communicating in the water, where the deepest depth is more than 0.025 m. (**b**,**c**) show how the robot climbed the step, and the height of the step was about 15 cm. (**d**) shows that the robot was traveling on the gravel ground. (**e**,**f**) shows how the robot climbed a slope, and the slope was about 26.

(1) Mobility: With the help of support legs, the robot could go up and down stairs and pass the barriers (shown in Figure 26b,c). The robot could climb about a 26° slope (shown in Figure 26e).
(2) Communication: The communication ability was tested when the robot was under remote telecontrol mode and semi-automatic mode. Including the enforceability and reliability of communication between the robots and OCU. The images, sounds, command messages and environmental information could be transmitted correctly.
(3) Battery supply capacity: All the components of the MSRBOTS are powered by the batteries, so the batteries’ capacity is an important factor. The batteries’ capacity for MSRBOTS is sufficient, and they could still supply the power for the robot system for about 5 h after the robot test for 3 h.
(4) Sensor system: During the test process, the cameras, audio, CD10 and CH${}_{4}$ lift sensor could keep working in the test room, which was full of CH${}_{4}$, safely. In the test, the video and audio worked normally, and the CH${}_{4}$ lift sensor and CD10 could obtain the gas information and transmit this accurately to the OCU via the communication system.
(5) Explosion-proof and waterproof ability: The robot could work successfully in the coal environment continuously in the test without any accident. The robot electric box and motor shells also passed the explosion test. Therefore the explosion-proof ability of the robot is very reliable. The water-proof ability was tested in a water pool, and the max water depth was about 0.03 m (shown in Figure 26a). The robot electric box and shells of the motors were sealed very well. Even though the sealing surfaces were submerged in water, the robot worked as normal when the robot traveled in the water pool.

### 6.2. Experiments in Tashan Coal Mine of Datong Coal Mine Group

**Figure 27:**
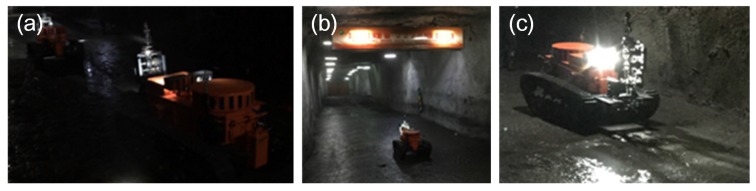
(**a**) shows that two robots are traveling in series in the coal mine in the semiautomatic control mode; (**b**,**c**) show that the robot is moving forward in the coal mine in the semiautomatic control mode with the $v_{f}$–$\Delta \theta_{f}$ method.

**Figure 28:**
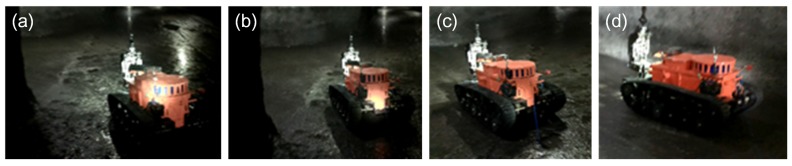
(**a**–**d**) show that a robot is turning left at a corner under the semiautomatic control mode with the $v_{f}$–$\Delta \theta_{f}$–*R* method.

**Figure 29:**
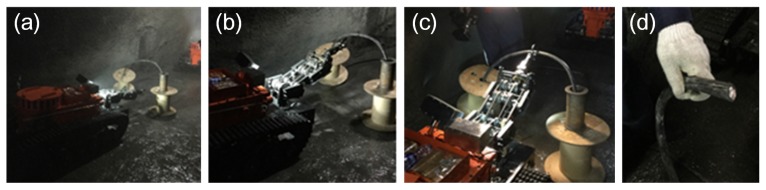
(**a**–**d**) show that a robot is cutting the cable controlled by OCU far away in the telecontrol control mode. (**a**) shows the robot is preparing to aim at the cable. (**b**) shows the robot has aimed at the cable. (**c**) shows the robot is cutting the cable. (**d**) shows the cable is cut by the robot.

The experiment environment in the coal mine is very different from working on the ground. The experiment results could be summarized as follows: (1) The robot could always travel on mud, coal, stakes and other unstructured ground. Although the electric compass could record the deflection angle of the robot, its movement trajectory was still not straight because the robot easily slipped on complicated ground. Therefore, the robot always stopped when it was close enough to the wall, so the control system of motion control unit was adjusted to make the robot deflect an opposite angle and continue going forward automatically when it was closed enough to the walls; (2) Two robots, under semiautomatic mode, communicated in series (shown in Figure 27a) and went straight into the coal mine smoothly (shown in Figure 27b,c). They also turned at corners successfully (shown in Figure 28a–c); (3) Both robots could cut out cables and steel roll bars in telecontrol mode (shown in Figure 29a–d). When the cables and roll bars were swaying, the operator would spend more time on adjusting the scissors to aim at them. When the steel wire ropes were very thin, it was hard for the scissors to cut out them unless they were tightened by moving the robot forward after the scissors caught them; (4) Since the relay box may slip when the ground was smooth, we added some hooks to it to hook the ground. Furthermore, the fiber-optic cable is strong enough, but must avoid beign tangled.

### 6.3. Training in Rescue Training Scenes of the Datong Brigade of National Mine Emergency Rescue

**Figure 30:**
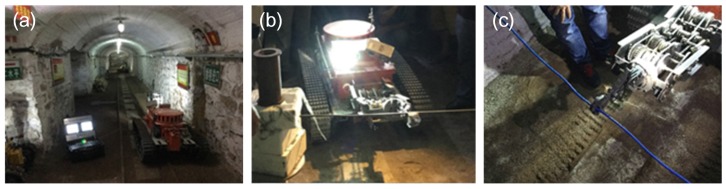
(**a**) shows the MSRBOTS in the simulated coal mine environment; (**b**) shows a robot cutting a steel roll bar; (**c**) shows a robot cutting a cable. The robot is controlled by OCU far away under the telecontrol control mode in both (b,c).

In Datong Brigade of National Mine Emergency Rescue, the rescuers were trained to use the MSRBOTS. The training contents included: (1) they are trained to operate the robot to move in the coal mine and to collect environmental information in a simulated coal mine; (2) the robot was controlled to move on unstructured ground with the rail (shown in Figure 30a); (3) the robot was operated to clean cables and steel roll bars by OCU under remote control mode (shown in Figure 30b,c). Finally, the rescuers were familiar with the robot system and gave us some advice, which is summarized as follows: (1) the robot is heavy and inconvenient to transport, and its weight should be reduced; (2) the rescuers advised that the robot should be assembled with more than 4 cameras to observe every perspective easily; (3) the robot should be much faster on the roadway to save rescue time; (4) they needed a much conciser and easier interactive interface for their understanding.

### 6.4. Results and Discussion

#### 6.4.1. Testing, Experiments and Training Results

In the coal mine, the speed of the robot could reach 1 m/s under remote control and could travel through water, coal, mud and other unstructured ground. Generally, the angle of slopes in the coal mine is lower than 16°, so the robot can travel in the coal mine smoothly. The fiber-optic cable box could release fiber-optic cable smoothly when robots move. The MSRBOTS could keep working in the coal mine for 8 h, including moving 2 h, and the batteries did not run out. The CD10, audio, cameras and CH4 lift sensor collected the environmental information precisely and could work normally in the coal mine. All data were transmitted to the OCU smoothly. The explosion-proof and waterproof functions of the robot were tested, and the test results showed that the robot could work in the coal mine environment safely.

#### 6.4.2. Discussion

Because of the complicated coal mine environment, the search-and-rescue robot and its manipulator needed to have their weight reduced to improve flexibility. The interactive interface of OCU should be concise for rescuers, and additional sensors should be added to the robot. However, if the new sensors were added in the robot, they must be intrinsically safe, and this is a challenge for the MSRBOTS.

## 7. Conclusions and Future Work

### 7.1. Conclusions

The MSRBOTS is developed in this paper for coal mine search-and-rescue, and it is composed of two explosion-proof and waterproof mobile robots and an OCU. We also discussed its mechanism, electrical system and experiments. The performances of MSRBOTS were also introduced based on tests and experiments. Its wading capacity, obstacle clearing ability and endurance capability are better than the majority of the robots of others, and the comprehensive performance is fine. Furthermore, it was approved by the Safety Approval and Certification Center. After many experiments, the MSRBOTS has proven that it can be used as a mobile sensor for rescuers to perceive the coal mine environments to make them much safer.

### 7.2. Future Work

Compared with other robots for coal mine environment sensing, the mobility of MSRBOTS, especially the passing ability for vertical obstacles, should be improved. The measures include mass reduction and optimizing the structure of traveling subassemblies. Because of the complicated coal mine environment, the robot is needs to have its volume reduced to improve its flexibility. The mechanical manipulator similarly should have its weight and geometry reduced. The interactive interface of OCU should be concise for rescuers or miners. The images obtained by the cameras should be used for assisting operators in driving the robot. Additional sensors should be added to the robot, such as 3D imaging devices used for obstacle avoidance and automatic path planing. However, if a sensor is used for the robot, the sensor must be intrinsically safe, and this is a challenge for the search-and-rescue robot system.

## Figures and Tables

**Table 1:** The general structure of the parameters of the robot.

Items	MSRBOTS	MINBOT II
Robot Body Sizes	1.315 × 0.536 × 0.862 m	0.82 × 0.589 × 0.747 m
Manipulator Length	0.664 m	No manipulator
Robot Mass	430 kg	130 kg
Environment Information	CH_4_, CO_2_ CO, O_2_, H_2_S, SO_2_, temperature, humidity, wind speed, air pressure	CH_4_, CO_2_, CO, O_2_, temperature, humidity
Audio	Two-way audio	Two-way audio
Video	Two cameras	Two cameras
OCU	0.5 × 0.4 × 0.25 m, 20 kg	No data

**Table 2:** General parameters of the manipulator. $m_{1}$ represents the mass of the shoulder joint; $m_{2}$ represents the mass of the wrist joint; $m_{3}$ represents the mass of the scissors joint; *m* represents the mass of the manipulator; *p* represents the location of the mass center; $T_{a}$ represents the maximum torque that the shoulder joint can bear; $\theta_{a}$ represents the motion range of the shoulder joint.

Items	Values	Units	Items	Values	Units
$m_{1}$	7.5	kg	*p*	0.361	m
$m_{2}$	7.5	kg	$T_{a}$	112.5	Nm
$m_{3}$	5.5	kg	$\theta_{a}$	−20∼90	°
*m*	20.5	kg			

**Table 3:** The MSRBOTS testing results.

Items	MSRBOTS	MINBOT II	Units
Height of vertical obstacle	0.15	0.5	m
Continuous steps	0.12 × 0.4	no data	m
Climbing angle	26	no data	°
Wading depth	0.3	no data	m
Ditch width	0.4	0.6	m
Communication distance	2000	1000	m
Power supply time	5	no data	h

## Abbreviations

The following abbreviations are used in this manuscript:

OCU	Operating control unit
BMS	Battery management system
IS	Infrared sensor
EC	Electronic compass
GY	Gyro
Δh	Increment height relative to current height
*h*	The current height of the scissors
$J_{1}$	Shoulder joint
$J_{2}$	Wrist joint
$J_{3}$	Scissors joint
$\Delta \theta_{pm}$	The angle by which the power motor needs to rotate
$\Delta \theta_{1}$	The rotation angle of the wrist joint
$\Delta \theta_{2}$	The rotation angle of the wrist joint
$L_{a}$	The length of the manipulator
$i_{J_{1}}$	Shoulder reduction ratio
$i_{J_{1}}$	Wrist reduction ratio
*R*	The radius of the robot when turning
$\Delta \theta_{f}$	The deflection angle relative to the current traveling direction
$v_{1}$	The speed of the left traveling subassembly
$v_{2}$	The speed of the right traveling subassembly
$v_{f}$	The speed of the robot
*b*	The distance between the left and right subassemblies
Δs	Tracks’ displacement
Δt	Moving time
